# Potentially Curative Therapeutic Activity of NEO212, a Perillyl Alcohol-Temozolomide Conjugate, in Preclinical Cytarabine-Resistant Models of Acute Myeloid Leukemia

**DOI:** 10.3390/cancers13143385

**Published:** 2021-07-06

**Authors:** Axel H. Schönthal, Steve Swenson, Radu O. Minea, Hye Na Kim, Heeyeon Cho, Nazleen Mohseni, Yong-Mi Kim, Thomas C. Chen

**Affiliations:** 1 Department of Molecular Microbiology & Immunology, Keck School of Medicine, University of Southern California, Los Angeles, CA 90089, USA; nmohseni@usc.edu; 2 Department of Neurosurgery, Keck School of Medicine, University of Southern California, Los Angeles, CA 90089, USA; sswenson@usc.edu (S.S.); minea@usc.edu (R.O.M.); heeyeonc@usc.edu (H.C.); 3 Department Pediatrics, Division of Hematology, Oncology, Blood and Bone Marrow Transplantation, Children’s Hospital of Los Angeles, Los Angeles, CA 90027, USA; hyekim@chla.usc.edu (H.N.K.); ymkim@chla.usc.edu (Y.-M.K.)

**Keywords:** cytarabine resistance, DNA alkylation, acute myeloid leukemia, perillyl alcohol

## Abstract

**Simple Summary:**

Many patients are still dying from acute myeloid leukemia (AML). Initial treatment of this blood-borne cancer consists of chemotherapy, usually with the agent cytarabine (AraC). However, the cancer cells can become drug resistant and unresponsive to AraC, which complicates further treatment and worsens prognosis. More effective treatments are needed. We are developing a novel anticancer compound called NEO212. We investigated its AML-therapeutic potential with the use of AraC-resistant AML cells grown in culture and in mice implanted with such AML cells. We found that NEO212 effectively killed AML cells in culture. The majority of AML mice that received NEO212 treatment survived and thrived without signs of tumor recurrence. At the same time, NEO212 treatment did not result in any detectable side effects, showing that this drug was very well tolerated by these animals. We deem it worthwhile to further develop NEO212 toward its evaluation in AML patients, in particular in those where initial therapy with AraC has failed.

**Abstract:**

Despite progress in the treatment of acute myeloid leukemia (AML), the clinical outcome remains suboptimal and many patients are still dying from this disease. First-line treatment consists of chemotherapy, which typically includes cytarabine (AraC), either alone or in combination with anthracyclines, but drug resistance can develop and significantly worsen prognosis. Better treatments are needed. We are developing a novel anticancer compound, NEO212, that was created by covalent conjugation of two different molecules with already established anticancer activity, the alkylating agent temozolomide (TMZ) and the natural monoterpene perillyl alcohol (POH). We investigated the anticancer activity of NEO212 in several in vitro and in vivo models of AML. Human HL60 and U937 AML cell lines, as well as different AraC-resistant AML cell lines, were treated with NEO212 and effects on cell proliferation, cell cycle, and cell death were investigated. Mice with implanted AraC-sensitive or AraC-resistant AML cells were dosed with oral NEO212, and animal survival was monitored. Our in vitro experiments show that treatment of cells with NEO212 results in growth inhibition via potent G2 arrest, which is followed by apoptotic cell death. Intriguingly, NEO212 was equally potent in highly AraC-resistant cells. In vivo, NEO212 treatment strikingly extended survival of AML mice and the majority of treated mice continued to thrive and survive without any signs of illness. At the same time, we were unable to detect toxic side effects of NEO212 treatment. All in all, the absence of side effects, combined with striking therapeutic activity even in an AraC-resistant context, suggests that NEO212 should be developed further toward clinical testing.

## 1. Introduction

Despite progress in the treatment of acute myeloid leukemia (AML), the clinical outcome remains suboptimal and many patients are still dying from this disease. AML progresses rapidly and becomes fatal within months if not treated. First-line treatment consists of chemotherapy, which typically is separated into an induction and consolidation (postremission) phase. The induction phase generally uses cytarabine (AraC) in combination with an anthracycline such as daunorubicin or high-dose AraC alone. After complete remission is achieved, composition of the subsequent consolidation therapy is variable and individualized based on prognostic factors, including tumor cytogenetics and general health of the patient, and may include further chemotherapy and stem cell transplantation. In addition, novel targeted therapies could be considered, such as the BH3-mimetic venetoclax, inhibitors of fms-like tyrosine kinase 3 (FLT3) or of isocitrate dehydrogenase 1 and 2 (IDH1, 2), and others. However, despite these complex therapeutic regimens, relapsed and refractory leukemia remains a challenge and better treatments are needed [1,2].

We are developing a novel anticancer compound, NEO212, that has shown promising activity in a variety of preclinical cancer models [3,4,5]. NEO212 was created by covalent conjugation of two different molecules with already established anticancer activity, temozolomide (TMZ) and perillyl alcohol (POH). TMZ is an alkylating agent commonly used for chemotherapy of malignant glioma and refractory anaplastic astrocytoma, and occasionally for metastatic melanoma and other cancers [6]. A 2002 Phase I study suggested that TMZ might have activity against relapsed/refractory acute leukemia [7], and later trials investigated its activity in combination with other agents, such as cisplatin or poly(ADP-ribose) polymerase (PARP) inhibitors, in similar patient populations [8,9,10]. However, TMZ can cause notable hematologic toxicity, in particular myelosuppression and rarely myelodysplastic syndrome and anaplastic anemia [11]. Unlike its standard use for malignant glioma, TMZ has not entered routine clinical practice for the treatment of leukemia.

POH is a naturally occurring monoterpene related to limonene that can be found in lavender oil, celery seeds, cranberries, cherries, and citrus fruit peel [12]. A large number of preclinical studies revealed its promising activity against a variety of cancer types. However, clinical Phase 1 and 2 trials using an oral POH formulation were unable to establish convincing therapeutic outcomes [13]. Currently ongoing clinical studies with recurrent glioblastoma patients are using a novel intranasal formulation to determine whether POH might show greater benefit when administered via the nose [14,15].

The rationale for conjugating POH to TMZ was based on our in silico studies indicating superior blood–brain barrier penetration of the NEO212 molecule as compared to either of its individual components [16]. While this prediction turned out to be correct [17], we further established that NEO212 exerted promising anticancer activity in a number of preclinical mouse tumor models, including glioblastoma, brain-localized breast cancer, melanoma, and others [3,4,5]. NEO212’s robust therapeutic impact prompted us to investigate additional tumor types with significant need for better therapies, which led us to AML. AraC is a key component of the standard first-line treatment regimen for this disease, but drug resistance and relapse occur in a substantial fraction of patients [18]. We therefore included several AraC-resistant AML cell lines in our study. In the following, with the use of preclinical in vitro and in vivo models, we present our evaluation of NEO212’s potential as a novel therapeutic drug for drug-resistant AML.

## 2. Materials and Methods

### 2.1. Pharmacological Agents

NEO212 was manufactured by Norac Pharma (Azusa, CA, USA) under current good manufacturing practice (cGMP) conditions and was kindly provided by NeOnc Technologies (Los Angeles, CA, USA). It was dissolved in DMSO (Santa Cruz Biotechnology, Dallas, TX, USA) at 100 or 500 mM for in vitro or in vivo experiments, respectively. Cytarabine (AraC; cytosine arabinoside) was purchased from Cayman Chemical (Ann Arbor, MI, USA) and dissolved in phosphate-buffered saline (PBS) at 2 mg/mL. Stock solutions of all drugs were stored at −80 °C. Upon thawing, drugs were diluted further with cell growth medium immediately before addition to cells. In all cases of drug addition to cells, the final DMSO concentration never exceeded 0.4% in the culture medium and was much lower in most cases.

### 2.2. Cell Culture

Human leukemia cell lines U937 and HL60 were obtained from the American Tissue Culture Collection (ATCC, Manassas, VA, USA). Cytarabine-resistant U937-derived cell lines 2C5, 4D9, and 6D10 were generously provided by David Largaespada’s lab (U. Minnesota, Minneapolis, MN, USA); they are described in Ref. [19]. All cells were propagated in RPMI medium supplemented with 10% FBS. Growth medium was supplemented with 100 U/mL penicillin and 0.1 mg/mL streptomycin. Penicillin, streptomycin, and RPMI (prepared with raw materials from Cellgro/MediaTech, Manassas, VA, USA) were provided by the Cell Culture Core lab of the USC/Norris Comprehensive Cancer Center. Cells were kept in a humidified incubator at 37 °C and a 5% CO_2_ atmosphere. FBS was obtained from Omega Scientific (Tarzana, CA, USA) and from X&Y Cell Culture (Kansas City, MO, USA). U937 and HL60 cells were passaged for less than 6 months after receipt, thus representing authenticated cells.

### 2.3. MTT Assay

Methylthiazoletetrazolium (MTT) assays were performed as detailed elsewhere [20]. Briefly, cells were seeded into 96-well plates at different densities ranging from 0.2–1.6 × 10^4^ per mL. Various concentrations of drug (or vehicle) were added and the cells were incubated for different lengths of time (48–144 h). For very long incubation times, we added 50% fresh medium at 72 or 96 h. AraC treatment was for 48 or 72 h, whereas NEO212 treatment was from 48 up to 144 h (due to slower cell death in response to this drug). At the end of the incubation period, MTT (Sigma Aldrich, St. Louis, MO, USA) was added for 4 h. In individual experiments, each treatment condition was set up in quadruplicate, and each experiment was repeated several times independently. Because all treatment conditions were applied to different cell densities and different lengths of incubation, we achieved consistent, reproducible results.

### 2.4. Cell Proliferation Analysis

Cell proliferation was assessed by counting cells over time. Independent cell cultures were exposed to different concentrations of NEO212. At different times, aliquots of cells were removed, mixed with Trypan blue, and counted in a hemocytometer. Blue cells were considered dead, whereas unstained cells were counted as live cells. Cell counts were independently repeated twice.

### 2.5. Analysis of Cell Cycle Distribution

We used the FxCycle^TM^ PI/RNase staining solution (ThermoFisher Scientific, Waltham, MA, USA), which contains propidium iodide (PI), DNase-free RNase A and a permeabilization reagent. After drug treatment, cells were collected, washed twice with PBS, and fixed with 75% ethanol. After storage at 4 °C for several days, fixed cells were washed twice to remove the fixative. After gentle centrifugation, the cells were suspended in 0.5 mL FxCycle solution and incubated at room temperature for 15–30 min in the dark. PI fluorescence, indicative of DNA content and cell cycle distribution, was determined by fluorescence-activated cell sorting (FACS) on a BD FACSAria II platform with FACSDiva^TM^ 8.0.2 software (BD Biosciences, San Jose, CA, USA) generating the histograms.

### 2.6. Cell Death Analysis with 7-AAD

After drug treatment, cells were washed with PBS, counted, and suspended in ice-cold PBS at 10^6^ cells/mL. A stock solution of 0.25 mg/mL 7-AAD (7-amino-actinomycin D) was prepared in DMSO, and 1 µL was added to 1 mL of cells. After at least 5 min of incubation, samples were loaded into a BD FACSAria II instrument (BD Biosciences, San Jose, CA, USA). Excitation was with a green laser at 561 nm, and emission was collected in the far red at 650 nm. Dot plots and histograms were generated for each sample, where the non-viable (i.e., 7-AAD positive) cells were counted relative to those in the untreated control sample.

### 2.7. Immunoblots

Total cell lysates were prepared and analyzed by Western blot as described previously [20]. We used the following primary antibodies—for the detection of cleaved caspase 3: monoclonal antibody (MAB10753) from MilliporeSigma, St. Louis, MO, USA) or monoclonal antibody (SC-271028) from Santa Cruz Biotechnology, Inc. (Dallas, TX, USA) and for PARP-1: SC-56196 from Santa Cruz (specific for the cleaved form) and #9542 from Cell Signaling Technology (Danvers, MA, USA) (recognizing full-length and cleaved PARP-1). Horseradish peroxidase-antibody conjugates (i.e., secondary antibodies) were obtained from Jackson ImmunoResearch Laboratories Inc. (West Grove, PA, USA). All antibodies were used according to the suppliers’ recommendations. For detection, SuperSignal West Pico PLUS Chemiluminescent Substrate was used (ThermoFisher Scientific, Waltham, MA, USA). Immunoblots were repeated to confirm the results. The uncropped Western blot images can be found at Figure S7.

### 2.8. In Vivo Experiments

All animal experiments were reviewed and approved by the Institutional Animal Care and Use Committee (IACUC) of the University of Southern California (USC). For the implantation of human tumor cells into mice, we purchased immune-deficient, female 6-8-week-old NOD-SCID or NSG-SGM3 mice from the Jackson Laboratory (Bar Harbor, ME, USA). For the determination of drug effects on blood and liver values, we obtained immune-competent, female 6–8-week-old Balb/c mice from the same source. The animals were housed at the USC Medical Center Animal Facility, which is AAALAC and AALAS certified and has written animal welfare assurance with the NIH-OLAW (Office of Laboratory Animal Welfare) that commits the institution to follow the standards established by the Animal Welfare Act.

For U937 cell implantation, we used NSG-SGM3 mice and intravenously (via tail vein) injected 5 × 10^4^ tumor cells in a volume of 200 µL 0.9% NaCl. For 4D9 and 6D10 cells, we used NOD-SCID mice. In addition, 50,000 4D9 cells were injected via tail vein in 50 µL of PBS. In a separate experiment, the same cell number and volume of 6D10 cells were injected into the peritoneum. Several days after tumor cell implantation, mice received treatment via oral gavage with 25 mg/kg NEO212 or vehicle only.

Non-tumor-bearing Balb/c mice were treated similarly. However, because initial toxicology results did not indicate any detrimental side effects after treatment with NEO212, we increased the dosage to 30 mg/kg and, instead of 5-day cycles followed by a treatment holiday, treatment was given once daily over a period of 28 consecutive days. Thereafter, animals were euthanized, and blood was collected by cardiac puncture. Further analysis (complete blood count with differential; Superchem blood chemistry panel) was performed by Antech Diagnostics (Fountain Valley, CA, USA).

### 2.9. Pharmaco-Analytic Measurements

After drug exposure, cells were rinsed twice with PBS and frozen as a “dry” cell pellet at −80 °C until processing. Upon thawing, cells were lysed with 200 µL acetonitrile and filtered with a 0.22 μm nylon filter (Nalgene, Rochester, NY, USA). High performance liquid chromatography (HPLC) was performed on an i-Series Plus Integrated HPLC System (Shimadzu, Columbia, MD, USA) with an integrated photo-diode array detector (PDA). LabSolutions V5.87 SP1 software (Shimadzu, Columbia, MD, USA) was used for data acquisition and instrument control. The isocratic separation of NEO212, TMZ, and AIC was performed using a Roc C18 column (10 mm × 4.6 mm × 3 μm) (Restek Corporation, Bellefonte, PA, USA) with a column temperature of 30 °C for 30 min. The isocratic mobile phase consisted of acetonitrile:phosphate buffer (38:62, *v*:*v*, pH 5). Flow rate was 1.0 mL/min. Ibuprofen (Cayman Chemical, Ann Arbor, MI, USA) was used as the internal standard. The analytes were detected with a diode array detector at 316 nm.

### 2.10. Statistical Analysis

All parametric data were analyzed using Prism 9 software (GraphPad Software, San Diego, CA, USA). Student *t*-tests were applied to calculate the significance values. Multi-group comparisons of blood and liver values were performed by Tukey’s multiple comparisons test. Kaplan–Meier survival probability analysis was done with log-rank (Mantel-Cox) test. A probability value (*p*) < 0.05 was considered statistically significant.

## 3. Results

**Figure 1:**
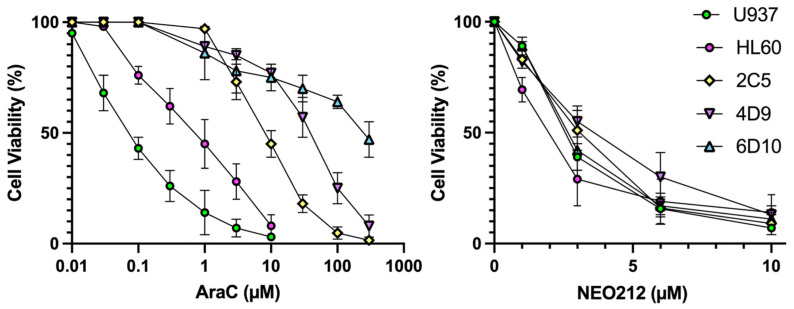
NEO212 is cytotoxic to AraC-resistant cells. U937, HL60, 2C5, 4D9, and 6D10 cells were exposed to increasing concentrations of AraC or NEO212, and cell viability was determined by MTT assay. Error bars represent SD from three replicates. Viability of untreated cells was set at 100% (there was no difference between vehicle-treated and untreated cells).

We characterized the anticancer effects of NEO212 on AML cells in vitro and in vivo with the use of established U937 and HL60 cell lines, as well as with well-characterized [19] AraC-resistant sublines of U937, named 2C5, 4D9, and 6D10. In vitro AraC sensitivity of all cells was confirmed and showed that 2C5, 4D9, and 6D10 cells had 126-fold, 629-fold, and 4043-fold increased IC50 values, respectively, as compared to AraC-sensitive parental U937 cells (Figure 1, Table 1). However, in response to NEO212 treatment, the cytotoxic IC50 remained in a very narrow range of 2.0–3.7 µM, revealing that all cell lines were similarly sensitive to NEO212, irrespective of their AraC resistance phenotype (Figure 1, Table 1).

**Figure 2:**
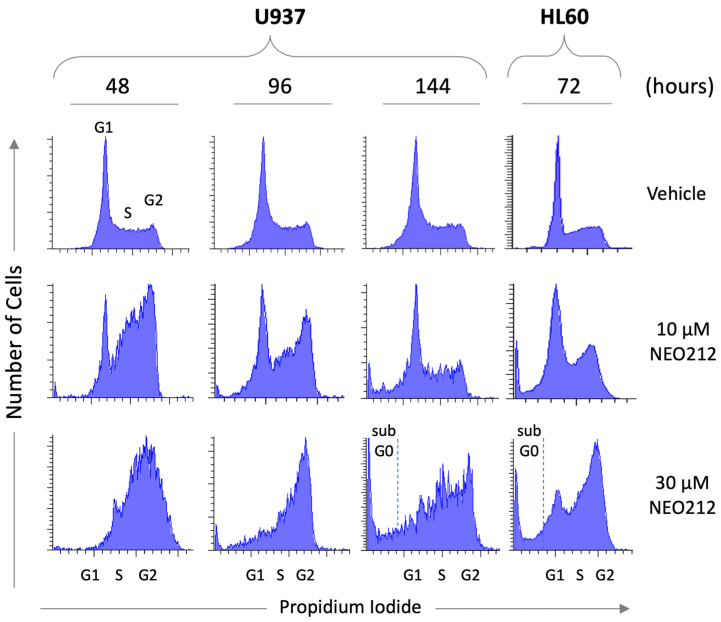
NEO212 triggers cell cycle arrest. U937 and HL60 cells were treated with 10 or 30 µM NEO212 or vehicle only. At different time points later, cells were collected and incubated with propidium iodide, followed by FACS analysis to determine cell cycle distribution.

To gain insight into the mechanisms that might underlie the potent inhibitory effect of NEO212, we subjected cells to NEO212 treatment and analyzed cell cycle distribution at different time points thereafter. Vehicle-treated control cells showed a typical G1-S-G2/M distribution of actively proliferating cells. In comparison, cells treated with 10 or 30 µM NEO212 revealed pronounced accumulation in G2/M (Figure 2). In the case of 10 µM, the G2/M block appeared to resolve between 96 and 144 h, which coincided with accumulation of cells in a sub-G0 peak. At 30 µM, the G2/M block did not resolve, although there was prominent accumulation of cells in G0 at 144 h.

The above analysis indicated growth arrest of viable cells, which was followed by slowly progressing cell death over several days. We further confirmed these events by counting the number of viable cells for up to seven days after a single treatment with increasing concentrations of NEO212 up to 40 µM. We found that all treated cells continued to proliferate for about 24–48 h, and then slowed down (Figure S1). At 5 µM, the cells eventually recovered after a lag time of 3–5 days and resumed proliferation. At 10, 20, and 40 µM NEO212, viable cells persisted for several days, but then their numbers declined to near-zero by 6–7 days. Microphotographs taken from these cells at 3, 5, and 8 days were consistent with these cell counts, picturing the demise of drug-treated cells over several days (Figure S2).

**Figure 3:**
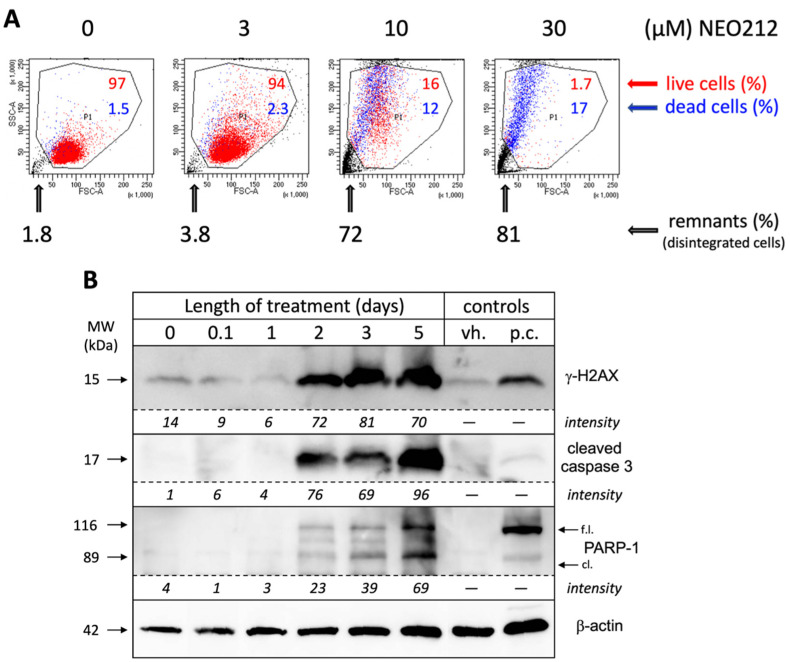
NEO212 causes apoptotic cell death. (**A**) 6D10 cells were incubated with 3, 10, or 30 µM NEO212. Control cells received vehicles or remained untreated. After eight days, cells were incubated with 7-AAD and analyzed by FACS analysis. Similar results were obtained with U937 cells; (**B**) U937 cells were exposed to 30 µM NEO212. At different time points, cells were harvested and total cell lysates analyzed by Western blot analysis for the indicated targets. Beta-actin was used as a loading control. Additional controls were vehicle-treated cells (vh.) and an unrelated lysate from staurosporine-treated cells that served as a positive control (p.c.). Signal intensities of NEO212-treated lanes were measured with ImageJ [21] and adjusted relative to corresponding actin bands.

In an effort to obtain a clear quantitative distinction between growth arrest and cell death, we incubated NEO212-treated cells with 7-AAD, followed by FACS analysis, which enabled the separation of dead cells, live cells, and remaining debris from disintegrated cells. As shown in Figure 3A, 10 µM NEO212 resulted in 16% live cells, 12% dead cells and 72% remnants, and 30 µM NEO212 only left 1.7% live cells, confirming the toxic impact of NEO212. To determine whether cell death was by apoptosis, we performed Western blot analysis of typical apoptosis markers, including cleaved caspase 3, cleaved poly[ADP-ribose] polymerase 1 (PARP-1), and phosphorylated H2A histone family member X (γ-H2AX), the latter an indicator of double-strand breaks that emerge during the apoptotic process. Figure 3B shows that all three markers were induced by NEO212, beginning at 48 h, and then further accumulated over the following three days. Overall, these data are consistent with apoptosis as the main process of cell death and further highlight the slow progression of these events over several days.

**Figure 4:**
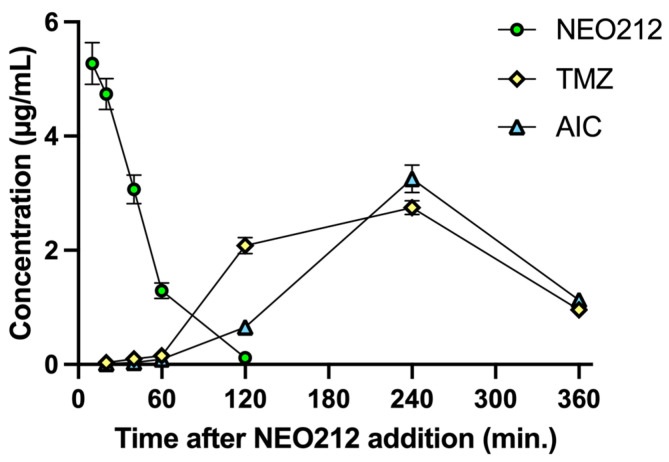
NEO212 treatment generates intracellular TMZ and AIC. HL60 cells were treated with 50 µM NEO212. At the indicated time points, cells were collected, extensively washed, and processed for pharmaco-analytic measurements of intracellular NEO212, TMZ, and AIC concentrations (mean ± SD, *n* = 3).

Tardy killing of tumor cells by NEO212 was reminiscent of the well-recognized [22] slow manner by which the DNA alkylating agent TMZ exerts its cytotoxic impact, i.e., requiring at least two cell doublings to enable its DNA methylation events to trigger apoptosis. TMZ is known to generate a highly reactive methyldiazonium ion (which is the species that methylates DNA), leaving behind 4-amino-5-imidazole-carboxamide (AIC) as an inert metabolite (see diagram in Figure S3). Because TMZ is a component of NEO212, we hypothesized that NEO212-induced cytotoxicity of AML cells might involve a similar mechanism. We therefore measured the intracellular amount of NEO212, TMZ, and AIC in HL60 cells treated with NEO212. Results presented in Figure 4 support our hypothesis. NEO212 is readily detectable inside cells but rapidly disappears within 2 h. In its stead, there is an increase first in TMZ and second in AIC, with a peak at 4 h and subsequent decline. Because the decay of TMZ and the presence of AIC is a clear indication that the reactive (very short-lived) methyldiazonium ion must have been generated, our data strongly support a model (Figure S3) where NEO212 alkylates DNA in the same manner as TMZ.

**Figure 5:**
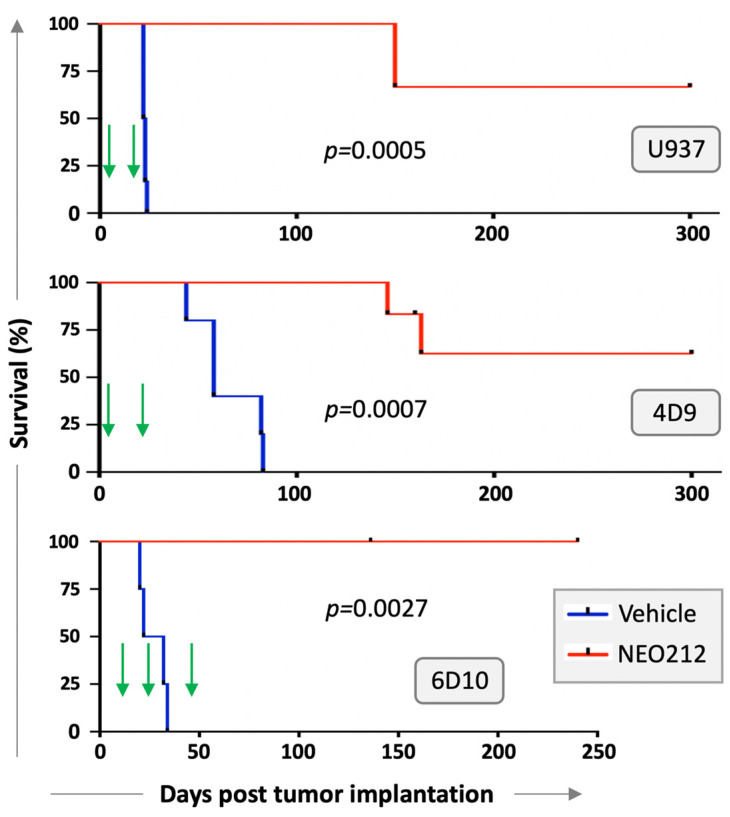
NEO212 exerts therapeutic activity in vivo. U937 cells were injected via tail vein into 12 immuno-deficient mice. Three days later, mice were randomly separated into two groups of 6 and treated via oral gavage with vehicle or NEO212. Treatment was once daily for five days (=1 cycle), followed by 10 days without treatment, followed by a second, final treatment cycle (the start of each 5-day cycle is indicated by a green arrow.) In a separate experiment, 4D9 cells were injected via tail vein into 10 mice. Three days later, mice were separated into two groups of five and treated as above, except that the treatment holiday between cycle 1 and cycle 2 was extended to 14 days. Furthermore, luciferase-labeled 6D10 cells were injected intraperitoneally into 10 mice, and tumor take was confirmed by bioluminescent imaging 11 days later. Treatment as above was started on day 11, with four mice in the vehicle group and five mice in the NEO212 group (one mouse was lost due to an accident). The first treatment cycle was followed by 10 days of treatment holiday. The second cycle was followed by 18 days of treatment holiday, and the final third cycle was administered on days 48–52. There were no further drug treatments after day 23 (U937 cells), day 27 (4D9 cells), or day 52 (6D10 cells). In all cases, survival of animals was monitored and is presented as Kaplan–Meier plots. *p*-values shown represent statistical difference between NEO212-treated and vehicle-treated groups.

We next investigated whether NEO212 would be able to exert anti-AML activity in vivo. Immuno-deficient mice were injected with U937 cells and treated with two cycles of NEO212 three days later. Each cycle consisted of 25 mg/kg oral NEO212 once a day for five consecutive days, and there was a non-treatment period of 10 days between the two cycles. There were no further treatments after the second cycle. Control mice received vehicle only. Kaplan–Meier survival graph in Figure 5 shows that all six vehicle-treated mice died within 26 days, whereas the six NEO212-treated animals survived significantly (*p* = 0.0005) longer: two mice died on Day 150, whereas the remaining four mice did not develop noticeable signs of disease and were still alive after 300 days.

We repeated modified setups of these experiments with AraC-resistant 4D9 and 6D10 cells. In the 4D9 model, we started the first cycle of NEO212 on Day 3 as before but provided an extended two-week treatment holiday before the second, final cycle. Survival is presented in Figure 5 and again shows a striking difference (*p* = 0.0007) between vehicle-treated (*n* = 5) and NEO212-treated (*n* = 5) animals. For the 6D10 model, we generated luciferase-positive cells, which were injected intraperitoneally, instead of via tail vein. Tumor take of 6D10 was confirmed by non-invasive imaging before initiation of treatment. Here, the first cycle was delayed and started only on Day 11; a total of three cycles were administered. Once again, vehicle-treated animals rapidly succumbed to disease (*n* = 4), whereas none of the NEO212-treated animals (*n* = 5) has shown signs of disease up to this point (currently: Day 240) (Figure 5). In summary, there was a robust, strikingly obvious increase in survival when animals were treated with NEO212, and many of the animals survived and thrived beyond the cutoff time of 300 days, suggesting a potentially curative outcome.

To assess the toxic impact of NEO212, we investigated several different parameters. For instance, we monitored body weight of all animals in all experiments. Treatment with NEO212 did not result in decreased body weight; instead, the mice continued to gain weight as expected. An example is shown in Figure S4.

**Figure 6:**
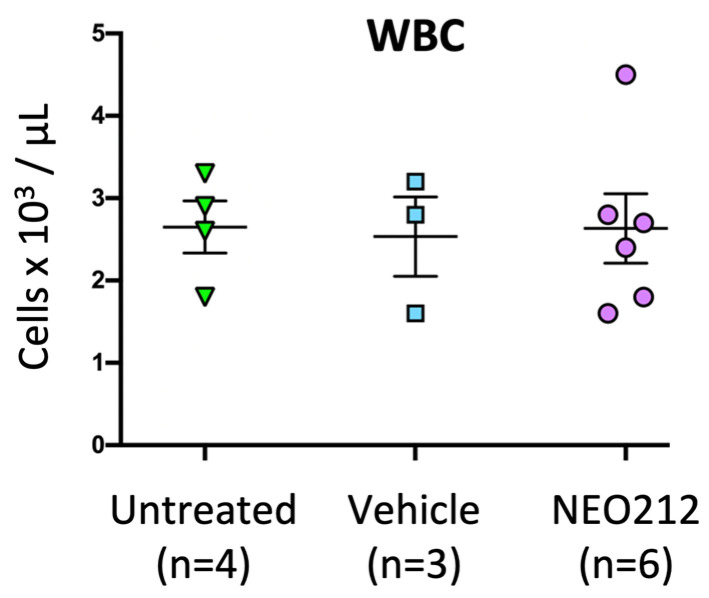
NEO212 does not cause leukopenia. Balb/c mice received 30 mg/kg NEO212 (*n* = 6) or vehicle (*n* = 3) via daily oral gavage for 28 consecutive days. Four (*n* = 4) animals remained untreated. At the end of treatment, blood was collected and subjected to CBC with differential. Shown here are numbers of leukocytes for each animal, along with mean ± SE. There was no statistically significant difference between the three groups; in all comparisons, the *p*-value was >0.98. Additional data from CBC analysis are presented in Figure S5.

Based on the presumed alkylating properties of NEO212, we also investigated leukopenia as a potential side effect. Immuno-competent Balb/c mice received one or two cycles of NEO212 at therapeutic dosages, and, immediately afterward, blood was drawn and subjected to complete blood count (CBC). However, no differences in white blood cell (WBC) count could be detected as compared to untreated animals. We therefore increased the dose to 30 mg/kg and extended once-daily treatment to 28 consecutive days, after which time blood was analyzed for CBC with differential, along with standard analysis of liver and kidney function. We used six mice for the treatment group and compared the results to data from four untreated mice and three mice that had received only vehicle on the same schedule. However, despite the substantially intensified treatment cycle—providing a 3.4-time higher overall drug exposure as compared to treatment with two cycles over a similar time period—there was no decline of WBC count in any of the treated animals (Figure 6). CBC with differential showed no serious decline of eosinophils, monocytes, neutrophils, lymphocytes or red blood cells, although a small decrease of hematocrit and hemoglobin levels were noted (Figure S5). Similarly, liver values remained within the normal range, with no changes in alkaline phosphatase and bilirubin levels, and only small increases in the blood levels of alanine aminotransferase and aspartate aminotransferase (Figure S6). A kidney panel was performed as well but did not reveal major changes in NEO212-treated animals (not shown). In all, we were unable to detect clear signs of toxic side effects of NEO212 treatment; instead, drug-treated animals continued to remain healthy and thrived.

## 4. Discussion

Our study employed preclinical AML models to investigate the potential therapeutic activity of NEO212, a novel hybrid molecule generated by conjugating POH and TMZ. In each in vitro and in vivo assay, NEO212 demonstrated profound anti-AML activity, including against highly AraC-resistant cells. The latter is particularly noteworthy because AraC represents a key component of current clinical care for AML, and treatment resistance is a common occurrence [1,2]. In three separate in vivo experiments with modified treatment parameters, single-drug NEO212 treatment of AML mice consistently and robustly resulted in long-term survivors, while, at the same time, we were unable to detect toxic side effects of drug treatment.

Long-term survival of NEO212-treated mice is particularly noteworthy because this outcome might point to potentially curative effects of the drug in some of these animals. In the case of U937 and 4D9 cells, the majority of animals continued to thrive until the pre-set endpoint of 300 days without any signs of disease; in the case of 6D10 cells, all of the NEO212-treated mice have survived to the current 240-day mark (experiment is still ongoing) and are thriving (Figure 5). This result is the more impressive in view of the fact that only two (U937; 4D9) or three (6D10) 5-day cycles of treatment were administered, and there was no further treatment between days 23–300 (U937), 27–300 (4D9) and 52–240 (6D10). In simplified terms, 300 mouse days are considered equivalent to 30 years of a human [23], which further underscores the impressive prolongation of healthy life by NEO212 in many of these animals. Although NEO212 has shown anticancer activity in other preclinical tumor models as well [3,4,5], our current study with AML models stands out due to the much greater, potentially curative, therapeutic impact of NEO212 in this disease model.

While our results point to the possibility of curative effects of NEO212, we have not yet investigated whether treatment was eradicative, e.g., whether perhaps any dormant leukemia cells might have persisted in the long-term survivors. While this scenario is possible, such cancer cells, if they indeed existed, clearly did not re-activate full-blown leukemia at later times (i.e., after 163 days), as no mouse fell ill beyond this time point. Furthermore, there seems to be room for further optimization of the NEO212 treatment schedule: three cycles of treatment appear to be better than two cycles of treatment—although limitations with small numbers of animals and different cell lines are acknowledged. Nonetheless, in view of the very well-tolerated nature of NEO212 treatment, it appears feasible to extend the number of cycles or dosages, if needed.

The bone marrow-suppressive side effects of many alkylation agents are well recognized and can represent a limiting factor during their clinical application. Our analysis of intracellular drug metabolism revealed (Figure 4) that NEO212 treatment of cells generated intracellular TMZ, a pro-drug that is well known for its DNA alkylating activity and is commonly used in the clinic for the treatment of patients with malignant glioma [24]. Moreover, we also measured intracellular AIC, which is the stable metabolite of TMZ that remains after the reactive DNA-alkylating methyldiazonium ion has been generated (see diagram Figure S3). All in all, these results were consistent with our previously published studies indicating that NEO212 exerts DNA alkylating activity [3,4,5]. Within this context, it was therefore unexpected to not find substantial detrimental impact of NEO212 on healthy blood cell counts, even after an intentionally intensified and prolonged treatment cycle that provided greater overall drug exposure than the two or three cycles that achieved striking therapeutic activity (Figure 6, Figures S5 and S6). Similarly, regular or dose-dense NEO212 treatments did not result in major changes in blood chemistry and liver/kidney values, although aspartate aminotransferase and alanine aminotransferase levels were slightly elevated. All in all, these readouts demonstrate that NEO212 was exceedingly well tolerated by these experimental animals and point to a rather wide therapeutic window that bodes well for considerations of future clinical testing and implementation.

As our results indicate, at least part of NEO212’s cytotoxic impact is based on DNA alkylation by its TMZ subunit. This raises the issue of the risk of treatment-related AML (t-AML), a rare condition where AML arises secondary to treatment of other tumors with alkylating agents [25]. In the context of relapsed and refractory leukemia, it appears that this risk is acceptable in view of the potential benefits that may be derived from certain alkylating agents. While some alkylating agents are not considered for applications in leukemia [26], TMZ is somewhat of an exception, due to its unique mode of cytotoxic action, which primarily is derived from methylation of O6-guanine—which represents the underlying reason for “tardy” cell death that stretches over several days [22] (and is consistent with our observations with NEO212: Figures S1 and S2). While t-AML has been reported in response to malignant glioma treatment with TMZ [27], it represents a rare event and has not discouraged leukemia trials where TMZ is used alone or combined with PARP inhibitors [8,10]. There are also occasional case reports where leukemia responded well to TMZ treatment (e.g., [28]). However, based on our prior studies on NEO212 [3,4,5], in conjunction with the data presented in this current report, we deem NEO212 superior to TMZ because it combines superb therapeutic activity with very low toxicity, at least in our preclinical models. While the underlying mechanism for this favorable therapeutic profile remains to be elucidated, we suggest that NEO212 should be developed further toward clinical testing.

## 5. Conclusions

Our preclinical study with AraC-resistant in vitro and in vivo models of AML characterizes NEO212 as an effective, well-tolerated novel therapeutic agent. This novel agent has the potential to improve prognosis especially for those AML patients with the greatest medical need, i.e., those where disease has become unresponsive to standard treatment with AraC-based regimens. For this reason, we deem it worthwhile to pursue development of NEO212 toward clinical testing in this patient population. Last but not least, its mode of oral administration would provide further advantages over many other AML drugs that must be given via intravenous infusion.

## Figures and Tables

**Table 1:** Average IC50 values and differential sensitivities to each drug are shown as fold increase over IC50 value of U937 cells.

Cell Line	IC50 AraC (µM)	Fold Increase over U937	IC50 NEO212 (µM)	Fold Increase over U937
U937	0.07	–	2.6	–
HL60	0.8	11	2.0	0.7
2C5	8.8	126	3.1	1.2
4D9	44	629	3.7	1.4
6D10	283	4043	2.7	1.0

Note pronounced AraC resistance of several cell lines, whereas all cells remained equally sensitive to NEO212.

## Data Availability

All data pertaining to this study are contained in the present article and its corresponding Supplementary Materials.

## Supplementary Materials

The following are available online at https://www.mdpi.com/article/10.3390/cancers13143385/s1, Figure S1: NEO212 delays proliferation at low concentrations and kills cells at higher concentration, Figure S2: NEO212 treatment results in slowly-progressing cell death, Figure S3: Projected intra-cellular breakdown of NEO212 in AML cells, Figure S4: NEO212 treatment of mice does not reduce body weight, Figure S5: Prolonged treatment with NEO212 does not result in leukopenia, Figure S6: Prolonged treatment with NEO212 does not result in severe liver damage, Figure S7: Uncropped Figure 3B.
