# Supplementary material for: Potentially Curative Therapeutic Activity of NEO212, a Perillyl Alcohol-Temozolomide Conjugate, in Preclinical Cytarabine-Resistant Models of Acute Myeloid Leukemia
Source: Cancers (Basel). 2021 Jul 6;13(14):3385. doi: 10.3390/cancers13143385 (PMC8305595; doi:10.3390/cancers13143385)
Supplement: Supplementary file 1 [file cancers-13-03385-s001.zip › cancers-1267890-supplementary.pdf]

cancers

MDPI

Potentially Curative Therapeutic Activity of NEO212, a Perillyl Alcohol-Temozolomide Conjugate, in Preclinical Cytarabine-Resistant Models of Acute Myeloid Leukemia

Axel H. Schönthal, Steve Swenson, Radu O. Minea, Hye Na Kim, Heeyeon Cho, Nazleen Mohseni, Yong-Mi Kim and Thomas C. Chen

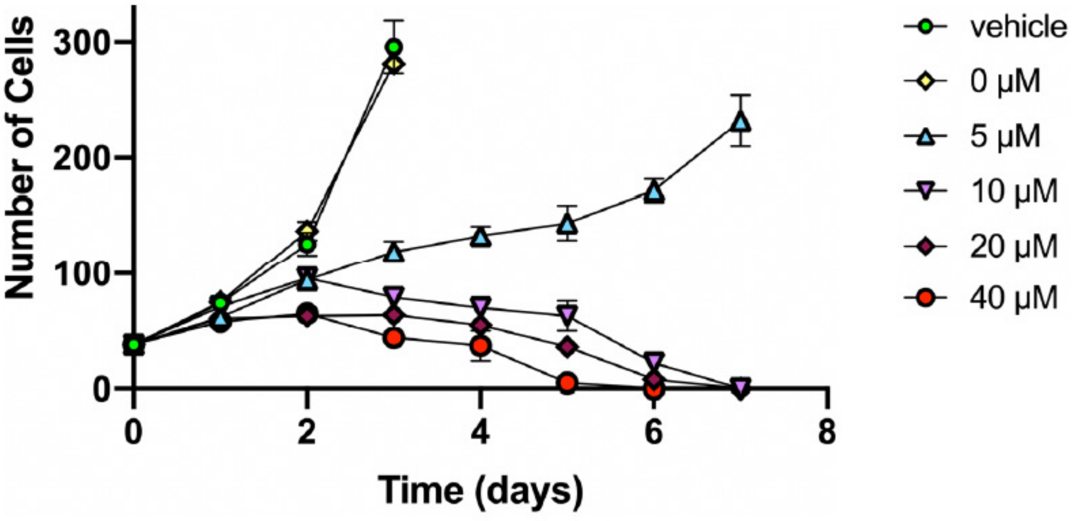

Figure S1. NEO212 delays proliferation at low concentrations and kills cells at higher concentration. U937 cells were seeded into 6-well plates and increasing concentrations of NEO212 were added at time 0. Every 24 hours thereafter, aliquots of cells were removed, and viable cells were counted *via* Trypan blue exclusion. Error bars represent SD from three independent replicates.

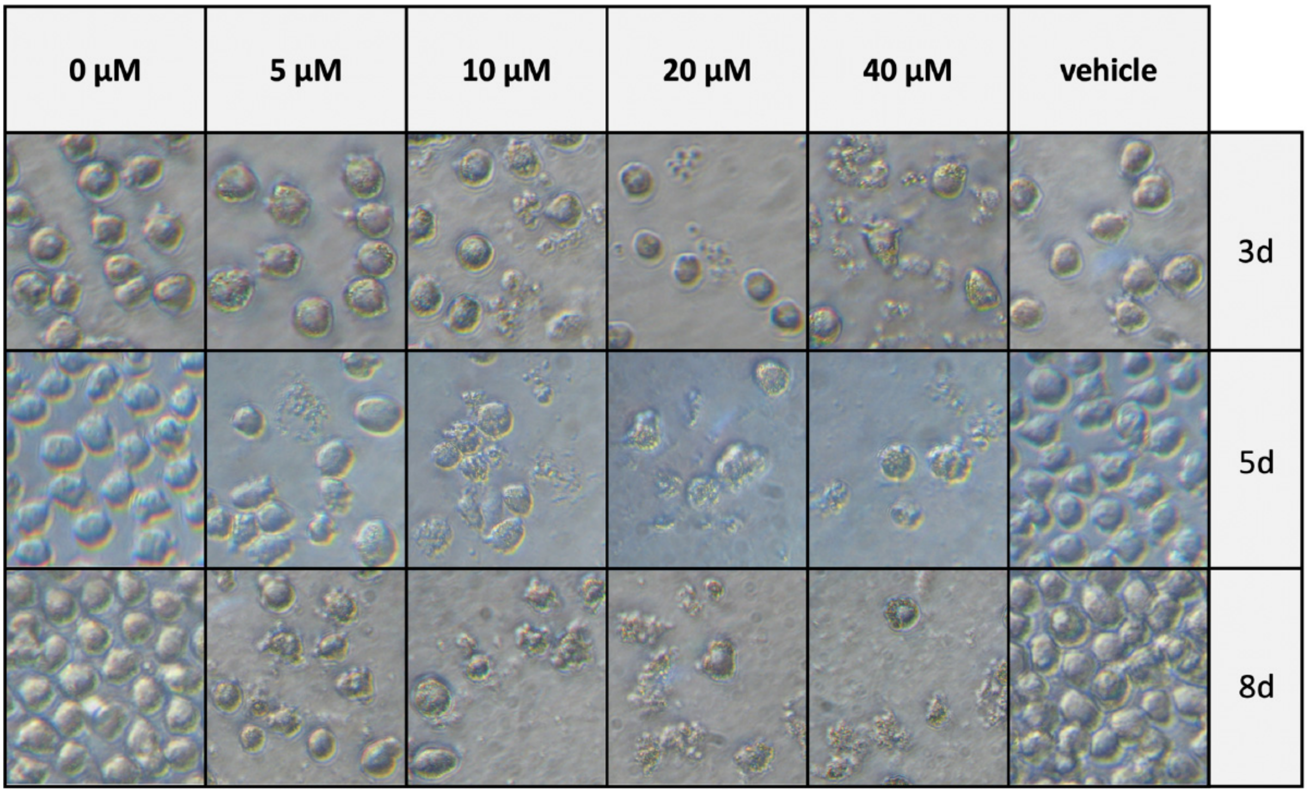

Figure S2. NEO212 treatment results in slowly-progressing cell death. U937 cells were seeded into 6-well plates and increasing concentrations of NEO212 were added at time 0. At 3, 5 and 8 days thereafter, microphotographs of cell cultures were taken. Representative photos are shown. After 3 days of treatment, viable cells were still visible (confirmed by Trypan blue exclusion) even at the highest NEO212 concentration; even after 5 days, occasional live cells were present. Dead cells and apoptotic bodies persisted.

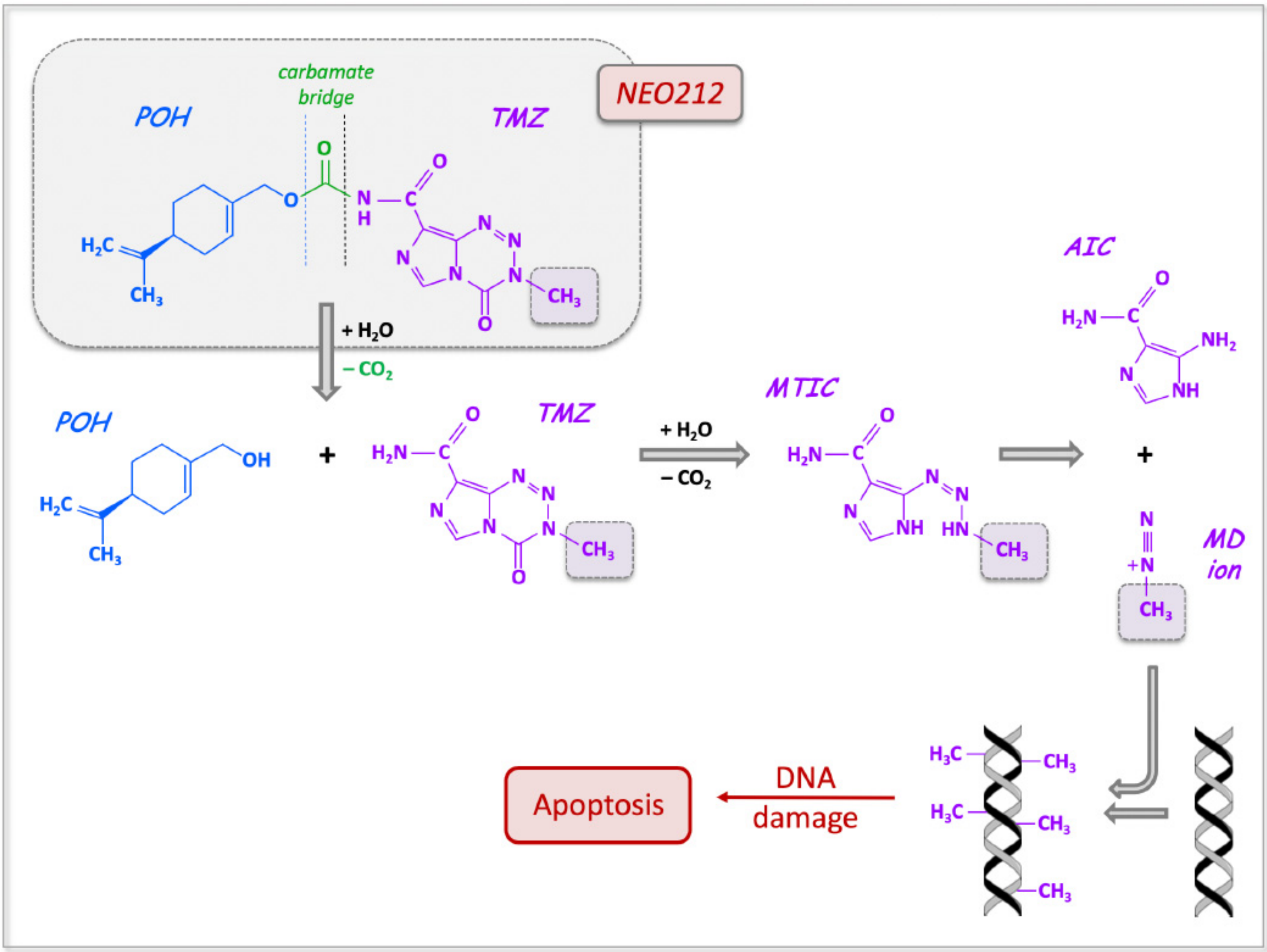

Figure S3. Projected intra-cellular breakdown of NEO212 in AML cells. Top left shows the intact NEO212 molecule, consisting of perillyl alcohol (***POH;* blue**) and temozolomide (***TMZ;* purple**) conjugated via a carbamate bridge (**green**). The small dashed square indicates the methyl group that will end up on the DNA molecule during the final DNA alkylation event.

The lipophilic POH moiety increases the efficiency of cell entry of NEO212. Inside the cell, hydrolases separate NEO212 into POH and TMZ. Under aqueous conditions and neutral pH, TMZ spontaneously decarboxylates (opening of tetrazinone ring) to generate 5-(3-methyltriazen-1-yl)imidazole-4-carboxamide (MTIC), which retains the DNA-alkylating methyl group. MTIC rapidly breaks down into 4-amino-5-imidazole carboxamide (AIC; a relatively stable metabolite) and the methyl-diazonium ion (MD), which is the reactive species that alkylates the DNA. Although a variety of lesions are set, it is methylation of O6-guanine (mO6G) that generally is critical for the drug's cytotoxic impact. Unless this methyl group can be efficiently removed by the cell's DNA repair system, it will result in double-strand DNA breaks after repeated, but futile rounds of repair during two consecutive cell cycles, which will trigger apoptosis.

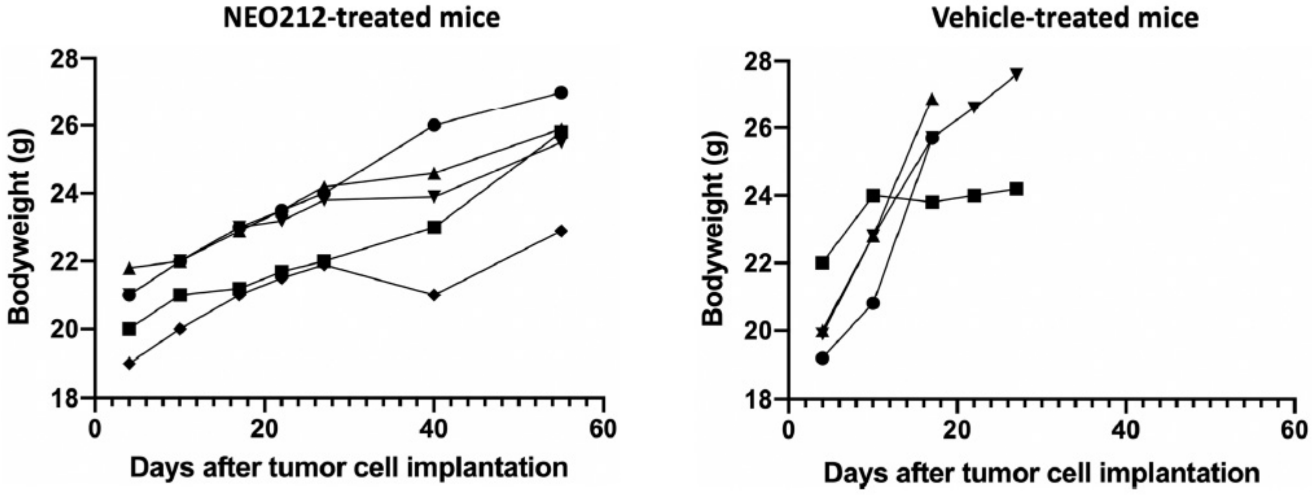

Figure S4. NEO212 treatment of mice does not reduce body weight. Among the indicators of toxic side effects of drug treatment is a reduction of body weight. We therefore measured body weights of all experimental animals in all our experiments. Shown here is one example from mice implanted with 6D10 cells. Both graphs present body weight of individual animals over time: n=5 for NEO212 treatment (left) and n=4 for vehicle-treated animals. In vehicle-treated animals, the increase in body weight over time was faster than what we usually observed in other experiments. We suspect that this might have been due to the intraperitoneal implantation of these cells, possibly resulting in ascites. Body weight increases shown for NEO212-treated mice were aligned with typical weight gain of healthy animals.

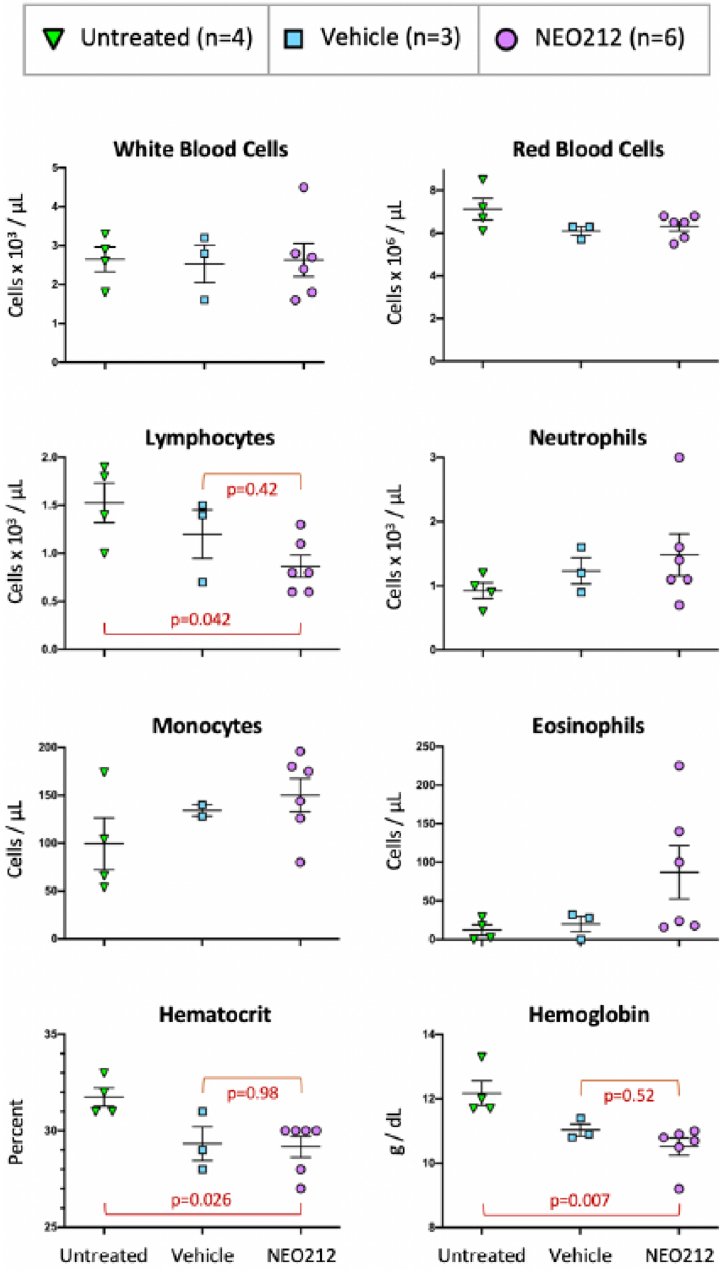

Figure S5. Prolonged treatment with NEO212 does not result in leukopenia. Six Balb/c mice received once-daily 30 mg/kg NEO212 for 28 consecutive days by oral gavage. Three mice received vehicle in the same manner. Four mice remained untreated. At the end of treatment, blood was drawn from all animals and subjected to complete blood count (CBC) with differential. *P*-values are shown only for those comparisons where the difference between untreated and NEO212-treated groups was <0.05. Although this was the case for "lymphocytes", "hematocrit" and "hemoglobin", the comparisons between vehicle-treated and NEO212-treated groups ***did not*** show significant differences (p>0.05) in these same targets, indicating only minor effects, if any, of NEO212 on these blood values.

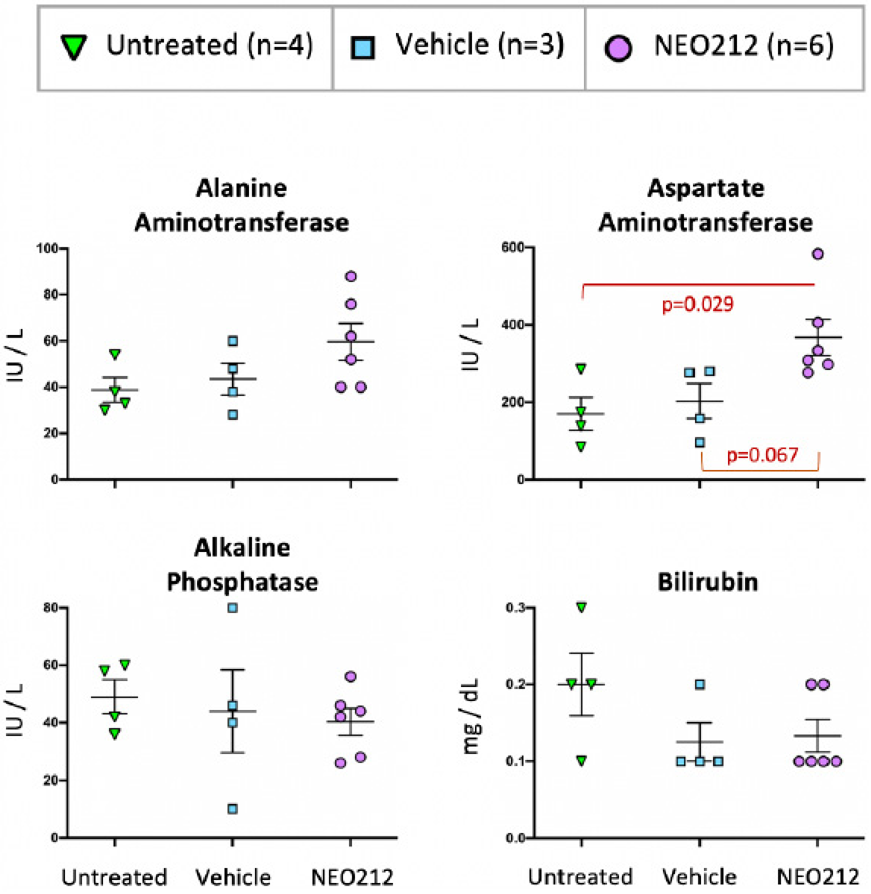

Figure S6. Prolonged treatment with NEO212 does not result in severe liver damage. Blood samples collected from mice described in the legend to Figure S5 were subjected to SuperChem test, which analyzes blood chemistry and provides information about the health of liver, kidney and pancreas. Shown here are liver values (kidney and pancreas values did not indicate toxic effects of NEO212 treatment). *P*-values are shown only in the case of aspartate amino-transferase, as this was the only target where the difference between untreated animals and NEO212-treated animals reached a *p*-value <0.05. However, comparison of vehicle-treated and NEO212-treated groups for this target ***did not*** show significant differences (*p*>0.05), indicating only mild impact, if any, of NEO212 on liver health.

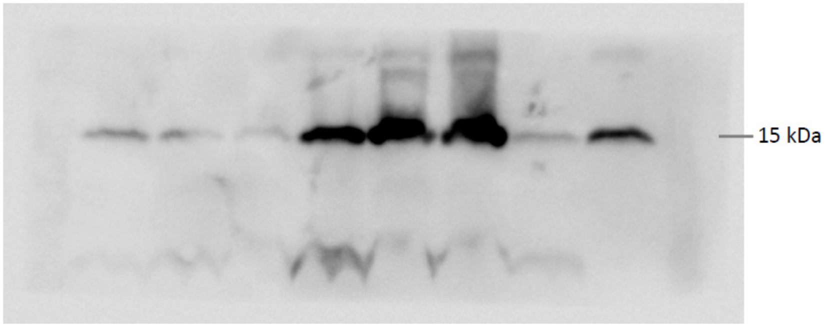

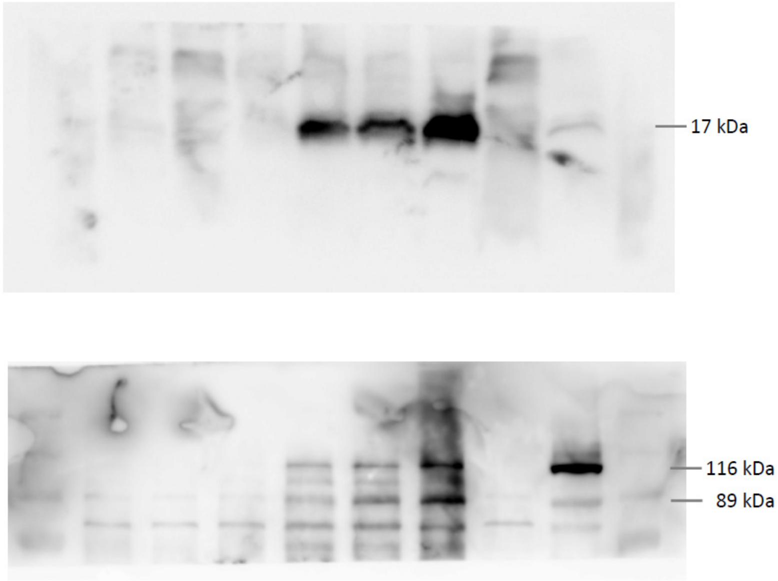

Figure S7. Uncropped Figure 3B.
